# Steps for implementing delayed cord clamping in a hospital setting

**DOI:** 10.1186/s40748-015-0011-8

**Published:** 2015-04-13

**Authors:** Ryan M McAdams, Carl H Backes, David J R Hutchon

**Affiliations:** Department of Pediatrics, Division of Neonatology, University of Washington, Box 356320, Seattle, WA 98195-6320 USA; Department of Pediatrics and Department of Obstetrics and Gynecology, Nationwide Children’s Hospital, The Ohio State University College of Medicine, Columbus, OH USA; Department of Obstetrics and Gynecology, Darlington Memorial Hospital, Darlington, UK

**Keywords:** Anemia, Birth, Newborn, Placental transfusion, Preterm

## Abstract

**Background:**

Delayed umbilical cord clamping (DCC) permits placental-to-newborn transfusion and results in an increased neonatal blood volume at birth. Despite endorsement by numerous medical governing bodies, DCC in preterm newborns has been slow to be adopted into practice. The purpose of this article is to provide a framework to guide medical providers interested in implementing DCC in a hospital setting. A descriptive implementation guideline is presented based on the author’s personal experiences and the steps taken at the University of Washington (UW) to implement DCC in premature newborns <37 weeks’ gestational age. Quality improvement data was obtained to assess compliance with DCC performance over the initial six months following initiation of the treatment protocol in July 2014. An anonymous electronic survey was administered to obstetrical providers in January 2015 to assess DCC policy awareness and adherence.

**Results:**

Important steps to consider regarding implementation of DCC in a hospital settings include applying a multidisciplinary educational approach aimed at motivating potential stakeholders potentially impacted by DCC, addressing safety concerns regarding DCC, and developing a standardized DCC treatment protocol. In the first month following DCC protocol implementation at UW, 79.2% (19/24) of premature newborns admitted to the neonatal intensive care unit received DCC, but compliance decreased over time, with DCC documented in only 40.1% (61/150) of newborns during the 6-month period following implementation. The majority of obstetrician survey respondents (90.9%, 20/22) were aware of the UW DCC policy for preterm deliveries, had performed DCC in the past 6 months (95.5%, 21/22), felt that they had sufficient understanding of the risks and benefits of DCC (90.9%, 20/22) and cited concerns for maternal hemorrhage and the need to resuscitate the baby as the main reasons to perform immediate cord clamping instead of DCC.

**Conclusion:**

Healthcare providers interested in implementing DCC may benefit from a procedural practice plan that includes an assessment of organizational readiness to adopt a DCC protocol, methods to measure and encourage staff compliance, and ways to track outcome data of infants who underwent DCC. Strategies to improve protocol awareness after DCC has been implemented are recommended since compliance may decrease over time.

## Background

At birth, delayed umbilical cord clamping (DCC) allows time for placental transfusion to the newborn and may provide benefits to both preterm and term infants compared to immediately cord clamping (ICC) [1-3]. The American College of Obstetricians and Gynecologists (ACOG) Committee Opinion has advocated DCC in preterm infants, when feasible [4]. Despite endorsements by numerous governing bodies (Table 1), including the World Health Organization [5], the American Academy of Pediatrics [4], the European Association of Perinatal Medicine [6], and the Society of Obstetricians and Gynaecologists of Canada [7], along with supporting evidence derived from 15 randomized control trials [1,2], the practice of DCC in preterm infants has been slow to be adopted, possibly due to anxiety during the period of delay and uncertainty about the ideal treatment choice and long-term outcomes [8-10]. The reluctance to adopt DCC in preterm infants suggests that guidelines describing how to promote implementation of DCC for these infants in a hospital setting may be valuable.

**Table 1:** **Recommended practice guidelines for delayed cord clamping** [4 **-** 6 **,** 30 **]**

	**Extremely Preterm**	**Preterm**	**Term**
	**<28 WGA**	**28–37 WGA**	**>37 WGA**
WHO	Delay of umbilical cord clamping for 1–3 minutes after birth is recommended for *all births* with simultaneous essential newborn care.		
ACOG	Evidence supports delayed umbilical cord clamping in preterm infants.		*Insufficient evidence* exists to support or refute the benefits of delayed umbilical cord clamping for term infants born in resource-rich settings.
AAP	Endorsed recommendations of ACOG (above)		
SOGC	Delayed cord clamping by at least 60 seconds is recommended		The risk of jaundice is weighed against the physiological benefits of delayed cord clamping.
RCOG	Delay clamping the umbilical cord earlier than necessary unless exigent circumstances such as heavy maternal blood loss or the need for immediate neonatal resuscitation take priority.		
ILCOR	Delay umbilical cord clamping for at least 1 min for newborn infants *not* requiring resuscitation. Evidence does not support or refute delayed cord clamping when resuscitation is needed.		

Abbreviations: *WHO* World Health Organization, *ACOG* American College of Obstetricians and Gynecologists, *AAP* American Academy of Pediatrics, *SOGC* Society of Obstetricians and Gynaecologists of Canada, *RCOG* Royal College of Obstetricians and Gynaecologists, *ILCOR* International Liaison Committee on Resuscitation, *WGA*, weeks gestational age.

In the United States, the practice of DCC in preterm newborns has not been widely adopted and few institutions have policies regarding DCC [11]. A myriad of barriers exist that may hinder adoption of DCC. Uncertainties surrounding DCC need to be addressed when considering implementing this practice. For provider’s accustomed to ICC, the recommended 30 to 60 second range to perform DCC in preterm infants [4] may be perceived as nonspecific and may raise concerns that inconsistent DCC practices could lead to variable outcomes. However, evidence-based guidelines are often collectively derived from studies that used non-standardized parameters, which leads to recommended ranges, exemplified in the Neonatal Resuscitation Program (NRP) with endotracheal tube epinephrine (1:10,000 concentration; 0.1 mg/ml) dose ranges from 0.5 – 1 ml/kg that can be repeated every 3 – 5 minutes [12]. Another barrier preventing endorsement of DCC may be provider perceptions of potential harm associated with DCC that may be incongruent with existing evidence refuting these concerns (e.g., the assumption that maternal hemorrhage is reduced with ICC when no difference has been demonstrated in multiple studies) [13]. A lack of awareness of potential infant benefits beyond placental neonatal transfusion, such as an almost 50% reduction in intraventricular hemorrhage all grades (10 trials, 539 infants, RR 0.59, CI 0.41-0.85, NNT 15) [2], may limit enthusiasm toward DCC. In addition, the term “delayed” cord clamping may also contribute to hesitancy in adopting DCC, since this term is often associated with a negative connotation of waiting, particularly in a society that values immediate service, instant access, and quick results.

Healthcare providers involved in delivery room care interested in implementing DCC may benefit from a procedural plan for incorporating DCC into their practice. Based on the author’s experiences with DCC and implementation of DCC for premature neonates (<37 weeks’ gestation) at the University of Washington (UW), we summarize a potential DCC implementation strategy. This process involves (1) applying a multidisciplinary approach to educate and motivate potential stakeholders that will be impacted by DCC, such as neonatologists, pediatricians, obstetricians, midwives, neonatal and obstetrical nurses, respiratory therapists, neonatology fellows, and pediatric and obstetrical residents, (2) addressing concerns regarding the safety and effectiveness of DCC, (3) developing a standardized DCC treatment protocol, (4) and establishing a method to measure staff compliance and to track outcome data for infants who underwent DCC. Here we describe a step-by-step overview (see Table 2) for implementing DCC that may apply to any hospital setting, but is most relevant to institutions performing high-risk deliveries of premature neonates.

**Table 2:** **Steps for implementing delayed cord clamping in a hospital setting.**

	**Potential Leaders**	
Recruit and Motivate	Obstetricians	Nurses
	Gynecologists	Nurse Practitioners
	Pediatricians	Respiratory Therapists
	Neonatologists	Midwives
	Trainees	Clinical Staff
Create	List of participants	Obtain input to resolve concerns
	Forum to describe DCC	
	Consensus to implement DCC	
	Specific DCC Protocol	
Implement	Teaching of DCC	Revise protocol as needed
	Simulations	
	Standard practices	
	Compliance monitoring	

## Results and discussion

Each of the authors has been involved with DCC at their respective medical institutions. In 2007, Darlington Memorial Hospital (author DJRH) was the first center in the United Kingdom to implement a DCC guideline (DCC of at least 30 seconds in preterm neonates and at least 45 seconds in healthy term neonates). In July 2014, DCC (45 seconds in preterm neonates) was initiated at UW, Seattle, WA, USA (author RMM), which had 2,010 hospital deliveries and 456 admissions to its 50 bed, Level 3 NICU in 2013.

From July to December 2014, a total of 230 neonates were admitted to the UW NICU, of which 150 were premature neonates <37 weeks’ gestational age. Among these premature neonates, DCC was documented (yes/no) in 70.7% (106/150) of neonates with DCC performed in 57.5% (61/106 of documented neonates; 40.7%, 61/150 of total NICU admissions) of these neonates. Documented premature neonates who had DCC performed compared to no DCC, had a mean gestational of 30.9 (±3, S.D) versus 32.4 (±3.9, S.D) weeks and a birth weight of 1.633 (±0.575, S.D) versus 1.906 (±0.777, S.D) kg, respectively.

To address factors pertaining to DCC policy compliance, a survey of obstetrical providers (attendings and fellows) involved in premature deliveries was conducted via email in January 2015, seven months after DCC was initiated. A total of 22 of 39 (56.4%) obstetrical providers (14/22 attendings, 3/7 fellows) responded to the survey. Most obstetrician survey respondents were aware of the UW DCC policy for preterm deliveries, had performed DCC in the past 6 months, and felt that they had sufficient understanding of the risks and benefits of DCC (see Table 3). Although the DCC policy was designed for preterm newborns, the 21 obstetrical provider survey respondents who performed DCC had done so in both preterm (8), term (1) and both preterm and term (12) newborns with DCC times ranging from 30 to >60 seconds, with most (63.6%, 14/22) adhering to the recommended 45 second delay. Obstetrical provider cited concerns for maternal hemorrhage, the need to resuscitate the baby, and requests by the neonatologist as the main reasons to perform ICC instead of DCC.

**Table 3:** **Answers given by obstetrical providers in response to survey questions regarding implementation and practices related to delayed cord clamping (DCC)**

**Questions:**	**Yes % (# yes, # no)**
Have you performed DCC in the past 6 months?	96% (21,1)
Do you feel that you have a sufficient understanding of the risks and benefits of DCC?	91% (20, 2)
Are you aware of the DCC policy for preterm newborns ≤37 and 0/7 weeks?	91% (20, 2)
In the past 6 months, are there preterm newborns ≤37 and 0/7 weeks that you intentionally opted for early cord clamping instead of DCC?	73% (16, 6)

### Initial steps in implementing delayed cord clamping

To promote evidence-based practice, a thorough review of current literature on DCC should be conducted with a focus on *Level 1* evidence (strongest), including systematic reviews or meta-analyses of all relevant randomized control trials (RCTs) and *Level II* evidence obtained from well-designed RCTs. After a formal decision on the part of hospital leaders involved in newborn care (e.g., director of the NICU or obstetrical service) to adopt DCC in clinical practice, implementation of this new practice requires planning and actions to modify currently established collective behavior in the pursuit of specific objectives (e.g., all premature newborns <37 weeks gestation will receive DCC for 45 seconds) [14]. The effectiveness of this clinical practice change, such as routinely practicing DCC instead of ICC, is dependent on the organizational readiness to change, a measure determined by factors such as behavioral, psychological, and structural preparedness [15].

#### Taking a leadership role in advocacy

A strong leader or cohesive team is required to champion the multi-step efforts required to successfully transition from well-established institutional practice that has little or no perceived drawbacks (e.g., ICC) and implement a new evidence-based practice like DCC. Klein and Sorra have described implementation as the critical gateway between the decision to adopt an innovation (e.g., DCC) and the routine use of the innovation within an organization, which occurs as a continuum, ranging from avoidance of the innovation to minimal, indifferent use to skilled, enthusiastic, and consistent use [16]. An individual or group of individuals interested in implementing DCC need to have a dedicated, patient, and persistent approach in order to overcome inertia common to established medical practices. By identifying key partners amongst healthcare providers involved in newborn deliveries, a leadership team can be established that serves as advocates of DCC and contact points for disseminating accurate and consistent recommendations as the DCC practice approach is rolled out.

#### Assess and address logistical and operational issues

Once a leadership team has identified a need to implement DCC based on current evidence-based literature and in accordance with recommended practice guidelines (Table 1), the team should determine how DCC would fit in with their institution’s system. The leadership team should assess the existing logistical and operational landscape pertaining to newborn deliveries. Key stakeholders (i.e., targeted users who are expected either to directly perform DCC or to support the practice) [16] and current resources relevant to newborn deliveries should be identified. Mapping out the existing institutional framework will aid in determining the essential units (e.g., obstetrical and neonatal intensive care), and staff (e.g., obstetricians, neonatologists, nurses, respiratory therapists, etc.) that will inform strategy development for making DCC operational. Additionally, potential hurdles should be identified (e.g., who will be responsible for announcing the DCC time at the delivery? Does the electronic medical record have current capacity to document DCC? Who will record DCC in the electronic medical record?) in order to explore possible solutions to implementation obstacles.

#### Educating and motivating stakeholders

The delivery of premature newborns is often a high-risk and intense endeavor that involves multiple healthcare providers to succeed. Successful implementation of DCC is contingent on the support of targeted stakeholders that will be impacted by DCC, including neonatologists, pediatricians, obstetricians, midwives, neonatal and obstetrical nurses, respiratory therapists, neonatology fellows, and pediatric and obstetrical residents. As an initial starting point, a Grand Rounds on DCC describing the physiology, background and evidence-based studies supporting this technique is a way to reach a large medical provider audience. Following this inceptive educational meeting, multiple interdisciplinary meeting sessions aimed at further education on DCC are recommended to address this diverse group of stakeholders. These sessions can provide an open forum to freely discuss concerns and questions about DCC. In order to realistically meet with the relevant stakeholders this approach requires flexibility with time and schedule coordination. Video streaming of live or recorded sessions on DCC is another method to reach medical providers unable to physically attend to a meeting. Contacting department or medical unit leaders early on may establish buy-in from key players and allow for discussion sessions on DCC to be included as agenda items during routine meetings (e.g., weekly obstetrical staff meeting, monthly respiratory therapist or neonatal nurse practitioner meeting, etc.). This strategy should increase the reachable audience and prevent the need to schedule additional meetings that may have poor turnout or provoke untoward sentiment (e.g., having to attend a meeting on a day off).

### Clarifying operational definitions

In order to safely and effectively implement DCC, providers involved in newborn deliveries need to have clear definitions of terminology and concepts related to DCC.

#### Time of birth

Clarification over when time of birth should be recorded is important when implementing DCC since this time point influences assignment of Apgar scores. Time of birth should be recorded and Apgar timing initiated once the fetus is delivered (complete expulsion or extraction from its mother) whether or not the umbilical cord has been cut or the placenta is attached. The available evidence suggests that DCC does not affect one or five minute Apgar scores, which may reassure providers used to performing ICC.

#### Immediate umbilical cord clamping after birth

Definitions of early cord clamping vary. In term infants, ICC is considered clamping the cord immediately or within the first 60 seconds after birth [13]. In preterm infants ICC is considered clamping immediately or within the first 30 seconds after birth [1]. The exact etiology of the common practice of ICC after delivery is not clear and not evidence based. As part of active management of the third stage of labor to reduce postpartum hemorrhage, ICC has been recommended along with a prophylactic uterotonic drug and controlled cord traction [17]. Subsequent studies have not demonstrated an effect of umbilical cord clamp timing (immediate or delayed) on postpartum hemorrhage risk [13].

#### Delayed umbilical cord clamping after birth

Similar to ICC, definitions of DCC vary. In term infants, DCC is considered clamping 60 seconds after birth, typically at 1 to 3 minutes after delivery [13]. In preterm infants DCC is considered clamping between 30 to 60 seconds after birth [1].

In healthy term singleton infants delivered in a hospital setting, placental transfusion of approximately 30-40% (24–32 ml/kg) of the total potential blood volume at birth typically occurs by 2 minutes after delivery, but may extend up to 5 minutes with infants held in a position level with the bed or raised to the level of the mother’s abdomen (vaginal births) or anterior thigh (caesarean births) [18]. In healthy, vaginally delivered term newborns, the position before cord clamping (i.e., maternal abdomen or chest) does not seem to affect volume of placental transfusion, a finding that supports early skin-to-skin contact with these newborns and their mothers [19]. Available DCC data on placental transfusion in preterm babies is based on positioning at the level of the mother’s abdomen (vaginal births) or anterior thigh (caesarean birth) with insufficient data regarding positioning preterm newborns at the level of maternal abdomen and chest, a position which may not be feasible in extremely premature newborns due to shortened umbilical cord lengths.

### Addressing concerns raised regarding delayed cord clamping

Although multiple RCTs have demonstrated the safety and potential benefits of DCC, reluctance to adopt this practice persists. Uncertainty about long-term outcomes requires additional adequately powered RCTs to establish long-term risks and benefits [9,10]. Like any recommended medical practice, as additional evidence becomes available, advice may change regarding optimal management of the umbilical cord at the time of delivery. While the lack of long-term outcome studies is acknowledged, many of the cited concerns for not performing DCC are not supported by RCTs. An example is the concern for symptomatic polycythemia resulting from DCC, which has not been demonstrated in multiple RCTs in preterm and term infants exposed to DCC compared to infants exposed to ICC [2,3,13].

Jaundice is another concern that may contribute to apprehension toward practicing DCC. While DCC has been associated with increased bilirubin levels in preterm [1] and term [13] infants compared to ICC, RCTs have demonstrated only a small increase (1.6%) in the need for phototherapy in term infants (not preterm) exposed to DCC based on data from 7 trials that included 2324 infants (RR 0.62 95% CI 0.41 to 0.96) [13]. Neither the meta-analysis by Hutton and Hassan nor the RCT by Andersson et al. showed a significant difference in the need for hyperbilirubinemia requiring phototherapy between full term infants managed with DCC versus ICC [3,20]. Kernicterus has not been reported as a complication associated with DCC. Given that most preterm infants are closely observed in the NICU for >48 hours, have bilirubin levels routinely measured, and effective phototherapy exists to safely treat hyperbilirubinemia, an over-emphasis on the increased risk of jaundice with DCC seems unwarranted when considering the potential benefits of DCC, particularly in high-risk neonates.

Concern has been raised that DCC will prevent timely resuscitation, particularly in premature newborns who appear cyanotic and/or apneic. During the first few minutes of age, cyanosis is common finding in premature newborns and should not discourage DCC. Whether apneic premature newborns would benefit from active resuscitation with positive pressure ventilation during DCC needs to be further studied. Although subjective, there have not been reported differences in Apgar scores and respiratory distress between infants exposed to DCC compared to ICC [3,13,21]. In comparison to ICC, DCC has not been associated with an increased risk of hypothermia after delivery [1]. On the contrary, Aziz et al. reported less hypothermia with infants who received DCC [22]. Emphasis should be made that DCC is a treatment aimed at promoting improved outcomes so that the time spent during DCC is not viewed as trivial, but propitious. While many of the benefits associated with DCC have not been studied as primary outcome measures, the decreased incidence of sepsis (2 trials, 137 infants, risk ratio 0.29, CI 0.09-0.99, NNT 10), necrotizing enterocolitis (5 trials, 241 infants, RR 0.62, CI 0.43-0.90, NNT 9), and intraventricular hemorrhage all grades (10 trials, 539 infants, RR 0.59, CI 0.41-0.85, NNT 15) are encouraging [2]. Two ongoing trials, the United Kingdom Cord Pilot Trial [23] and the Australian Placental Transfusion Study (The Australian New Zealand Clinical Trials Registry; https://www.anzctr.org.au) will hopefully clarify the long-term neurodevelopmental impact of DCC in preterm infants <32 week’s gestation. A discussion with parents prior to delivery on the potential risks and benefits of DCC may be beneficial and should be considered when feasible.

### Potential contraindications to delayed cord clamping

Interdisciplinary discussions between neonatology and obstetrical providers should include potential contraindications to DCC. While evidence based medicine recommendations on absolute contraindications to DCC do not exist, there are common conditions that may warrant ICC or umbilical cord milking as an alternative to DCC [24,25]. Circumstances that may not be ideal for DCC include cord prolapse, antepartum hemorrhage, fetal compromise in a multiple gestation pregnancy, and concern for meconium aspiration. Clear communication mechanisms (e.g., call outs) should be developed that allow obstetrical or neonatology team providers who have safety concerns before or during delivery to forgo DCC and provide immediate resuscitation.

### Creating a delayed cord clamping treatment protocol

To help ensure homogeneity of practice, a standardized DCC treatment approach is recommended. Developing a simple, easy to follow, and evidence-based treatment guideline should promote clinician acceptance and improve adherence. An example of a DCC protocol that can be used at the time of delivery is demonstrated in Table 4. As new information becomes available from clinical trials on DCC, the leadership team or DCC oversight committee can modify guidelines as needed to ensure that safe and optimal treatments are practiced in newborns. Similar to updates with NRP, disseminating new or altered recommendations on DCC will require scheduled educational sessions and compliance monitoring.

**Table 4:** **Delayed Umbilical Cord Clamping (DCC) Protocol**

1.	Prior to delivery, establish a consensus that cord clamping will be delayed for a specified duration (range 30–60 sec).
2.	Prepare two warm sterile towels for transfer of the infant from the obstetrician to the neonatologist.
3.	An assigned timekeeper starts a timer as soon as the infant is delivered from the womb, and thereafter announces the time in 15-second intervals.
4.	DCC: Upon delivery, the infant is held in the warm towel by the obstetrician and cord clamping is delayed for the specified interval.
5.	When the delay interval has been reached, the obstetrical provider clamps the umbilical cord in standard fashion and calls out “Cord clamped!”
6.	During the DCC interval, it is appropriate to call out any possible safety concerns as they may arise.
7.	The infant is transferred to the neonatologist’s warm towel and routine newborn resuscitation is performed per current NRP guidelines.
8.	The duration of DCC is recorded in the electronic medical record.

### Evaluating readiness

Successful implementation of a new practice, such as DCC, requires leaders and staff members with motivational readiness (i.e., a perceived a need for change from ICC to DCC) and a favorable organizational climate (e.g., clear goals, open communication, a cohesive staff, and willingness to change) [26]. Given the simplicity of practicing DCC, the need for additional institutional resources (e.g., increased staffing levels and physical resources) is limited. After the essential components to DCC have been mapped, potential hurdles addressed, key stakeholders educated, and the operational definitions established, then the leadership team needs to determine when to initiate the practice of DCC. Survey tools exist to measure organizational readiness for implementation of evidence-based practice into routine clinical care and may be useful when evaluating readiness to implement DCC [15].

#### Simulation exercises to promote confidence

Prior to actually performing DCC in the delivery room, healthcare providers may benefit from simulation-based training scenarios highlighting important steps in the conducting DCC in newborn infants. Similar to programs like NRP, a simulated delivery scenario that includes DCC may provide a safe learning environment that enhances provider’s development of critical leadership, communication and teamwork skills related to DCC. An aim of the simulated scenario would be to establish an effective working model that boosts the confidence of educated stakeholders and promotes familiarization with the steps involved in the DCC protocol. Simulation-based DCC training scenarios could be conducted jointly with different disciplines (e.g. neonatal and obstetrical providers) to promote effective communication, practice techniques, and strategies targeting teamwork competencies and learning objectives [27].

### Documentation and monitoring outcome data

Systematically documenting the duration of DCC in the medical record and routine tracking of outcome data in infants who underwent DCC is important in order to assess the ongoing benefits and potential risks of this practice. Safety and benefit has been consistently demonstrated in the available DCC trials for both term newborns in resource-poor settings and preterm babies (28–37 weeks’ gestation) not needing active resuscitation. However, there is limited outcome data pertaining to DCC in term newborns born in resource-rich settings or premature newborns (<28 weeks’ gestational age) needing resuscitation after birth. While future randomized control trials are needed to determine certain primary outcome measures, gathering accurate data will provide a framework to investigate associations with DCC and short- and long-term outcomes that may lead to practice modification and quality improvement. Disseminating this valuable information on DCC implementation and outcomes at meeting, through online discussion forums, and through scholarly publications will promote an evolving best-practice that should further benefit preterm infants.

**Figure 1:**
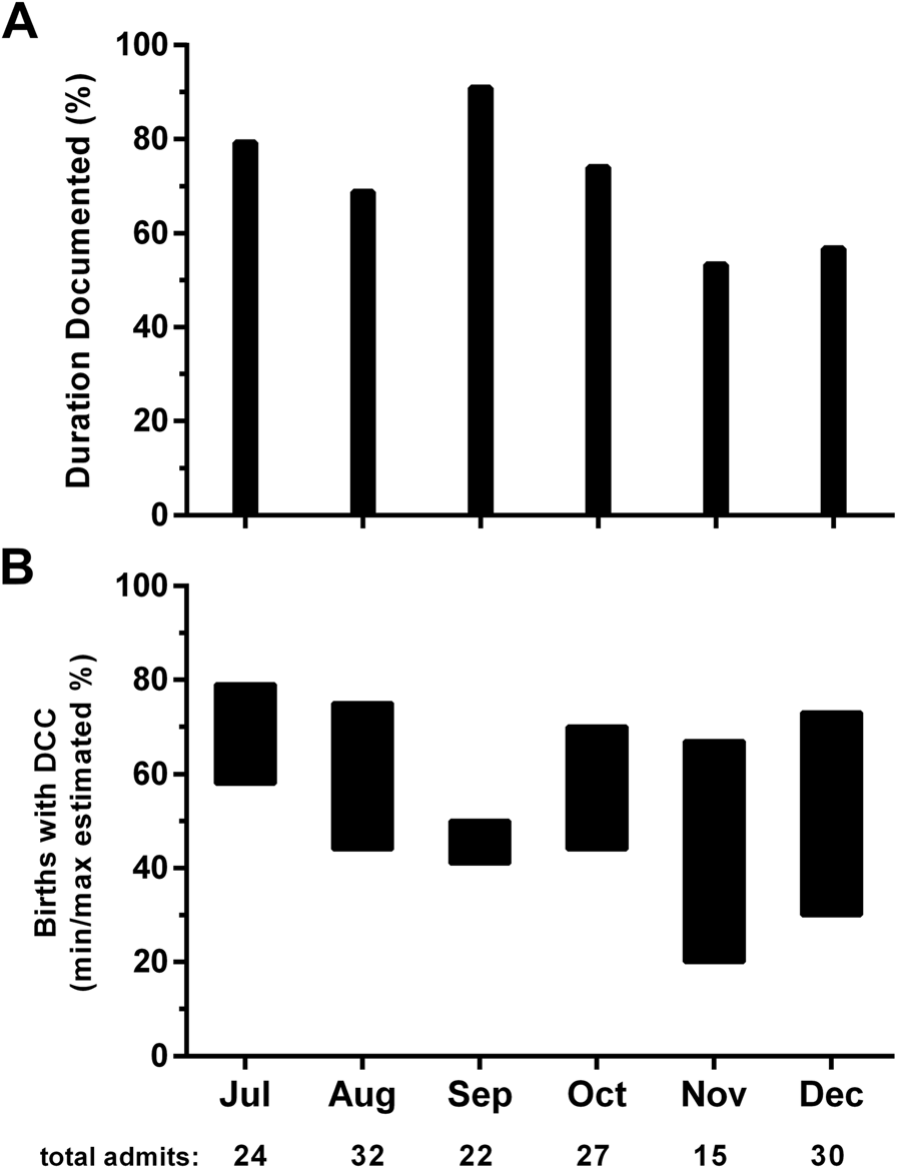
**Quality improvement assessment of compliance to performing and documenting delayed cord clamping in premature newborns.** Between July –December 2014, 150 premature neonates (<37 weeks’ gestational age) admitted to the University of Washington neonatal intensive care unit were eligible for delayed cord clamping (DCC), which was implemented in July, 2014. **A)**. For each month following DCC implementation, documentation (percent) was assessed regarding whether or not the duration of DCC was documented in the electronic medical record. Of the 150 neonates admitted to the NICU, 70.7% (106/150) had documentation on duration of cord clamping (delayed or immediate), with a decrease in documentation noted the last 2 months assessed. **B.)** Over the first 6 months following implementation, only 40.7% (61/150) of premature neonates admitted to the NICU had DCC (>30 seconds) documented. The figure depicts the neonates with documented DCC for each month (bottom line of the rectangular black bar) and the possible range of neonates who may have received DCC if all the undocumented neonates actually had DCC performed (top line of the rectangular black bar).

Periodic auditing medical records may provide a method to assess proper compliance and documentation of DCC. An audit of UW NICU admissions following the first six months after implementation demonstrated that only 70.7% (106/150) of premature newborns had duration of cord clamping documented in the electronic medical record, with only 40.7% (61/150) of premature newborns receiving DCC (>30 seconds) as demonstrated in Figure 1. Further analysis demonstrated that both documentation of cord clamping duration and performance of DCC decreased overtime. While the cause for this decrease may be multifactorial, one potential reason may be related to an inconsistency in the person(s) responsible for documentation. At UW, pediatric resident physicians are responsible for DCC documentation in the medical record and new residents that switch NICU rotations monthly may not be familiar with their obligation for data entry. These findings prompted measures to remind and re-educate providers involved with DCC on the existing policy and prompted inquiries about any reasons for DCC noncompliance. For example, as a measure to promote improved DCC documentation in the electronic medical record, the data entry field for documenting DCC was modified (e.g., highlighted in red as a required data entry box).

While the success rates for implementing changes in health care organizations are not clear, published estimates indicate that success rates range from 19% to 58% for businesses implementing various changes (e.g., changes in technology, strategy deployment, and culture) [28]. Our data regarding a new cord clamping duration policy are consistent with this relatively low success rate based on the documented DCC compliance rate of 40.7% in the first 6 months following policy initiation. Azziz et al. noted that a reason for noncompliance in practicing DCC included the neonatal team not having arrived at the delivery in time [22]. In our study, OB providers at UW cited concerns for maternal hemorrhage, the need to resuscitate the baby, and requests by the neonatologist as the main reasons to perform ICC instead of DCC. While the majority of obstetrical survey respondents were aware of the UW DCC policy for preterm deliveries and had performed DCC in the past 6 months, only 56.4% (22/39) of obstetrical providers responded to the survey. This incomplete survey data, similar to the deficient DCC documentation in the electronic medical record, underscores some of the challenges associated with implementing a new practice. As the UW DCC implementation experience illustrates, collecting partial data prevents an accurate assessment of actual adherence to a newly introduced DCC policy. In order to assess the impact of DCC on neonatal outcomes, the degree to which the DCC strategy is performed as intended by the leadership team who developed the implementation strategy (strategy fidelity) needs to be measured [29]. If DCC is not being practiced as intended, it is not possible to draw valid conclusions about the effectiveness of this strategy.

To promote compliance to new healthcare practice policies, a committed leadership team is needed to address issues and recommend effective solutions. Training and educating new staff on DCC is another essential step in maintaining a consistent, safe, and effective approach to DCC. Finally, high quality communication between delivery and stabilization teams is paramount for DCC to be successful. The most common comment by obstetrical providers surveyed at UW was that a reminder to perform DCC would be helpful either at a morning huddle discussing potential deliveries or at a “time out” prior to a delivery. A brief conversation prior to delivery and a post-delivery debrief may help achieve the goal of augmenting placental transfusion at the benefit of the preterm newborn.

## Conclusions

Implementation of DCC in a hospital setting requires a dedicated leadership team to educate and motivate key stakeholders to successfully modify the existing practice of ICC. Healthcare providers interested in implementing DCC may benefit from a procedural practice plan that includes an assessment of organizational readiness to adopt a DCC protocol, methods to measure and encourage staff compliance, and ways to track outcome data of infants who underwent DCC. Strategies to improve DCC implementation effectiveness, such as regularly promoting DCC protocol awareness, are recommended since compliance may decrease over time. Ongoing monitoring of DCC outcomes is essential to determine the long-term risks and benefits of the practice.

## Methods

The author’s opinions on how to safely implement DCC in a hospital setting are based on personal experiences with DCC at their respective tertiary care institutions. The recommended DCC implementation strategy constitutes the actual steps taken at UW with conclusions derived from this process. A DCC policy was initiated for all premature infants born at <37 weeks’ gestational age in July 2014 following a 17-month long development and education phase as part of the implementation process. As part of the policy, the goal duration of DCC was 45 seconds, a time delay chosen in accordance to the evidence-based recommended 30–60 delay advocated by the ACOG Committee Opinion practice statement, which is endorsed by the American Academy of Pediatrics [4]. For term deliveries, DCC is not routinely performed at UW, but is done upon parental request, with a delay of 1 to 3 minutes, a duration based on available evidence [13].

As a quality improvement measure, a review of UW neonatal intensive care unit (NICU) admissions from July to December 2014 was conducted to assess adherence to the DCC policy in preterm neonates. As an additional quality improvement effort, a survey of obstetrical providers (fellows and attending physicians) involved in newborn deliveries was conducted in January 2015 to assess awareness and attitudes toward the DCC policy. An exempt status was sought and granted by the UW Institutional Review Board for publication of the de-identified, minimal risk quality improvement data.

## Abbreviations

DCC: Delayed cord clamping
ICC: Immediate cord clamping
NICU: Neonatal intensive care unit
